# 2,3-Dimethyl-*N*-[(*E*)-(1*H*-pyrrol-2-yl)methyl­idene]aniline

**DOI:** 10.1107/S1600536810031867

**Published:** 2010-08-11

**Authors:** M. Nawaz Tahir, Muhammad Ilyas Tariq, Shahbaz Ahmad, Muhammad Sarfraz, Riaz H. Tariq

**Affiliations:** a Department of Physics, University of Sargodha, Sargodha, Pakistan; b Department of Chemistry, University of Sargodha, Sargodha, Pakistan; c Institute of Chemical and Pharmaceutical Sciences, The University of Faisalabad, Faisalabad, Pakistan

## Abstract

In the title compound, C_13_H_14_N_2_, the dihedral angle between the aromatic rings is 69.73 (14)°. In the crystal, inversion dimers linked by pairs of N—H⋯N hydrogen bonds generate *R*_2_^2^(10) loops. A weak C—H⋯π inter­action also occurs.

## Related literature

For background to Schiff bases and for related structures, see: Hussain *et al.* (2010*a* ▶,*b* ▶), Sarfraz *et al.* (2010 ▶); Tariq *et al.* (2010 ▶): For graph-set notation, see: Bernstein *et al.* (1995 ▶).
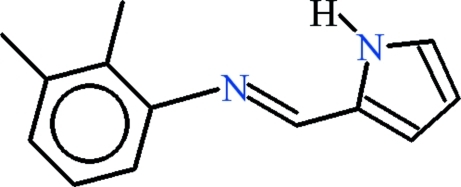


## Experimental

### 

#### Crystal data

C_13_H_14_N_2_
*M* *_r_* = 198.26
Monoclinic, 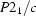

*a* = 12.8684 (9) Å
*b* = 7.1649 (5) Å
*c* = 12.9517 (9) Å
β = 107.613 (3)°
*V* = 1138.18 (14) Å^3^
*Z* = 4
Mo *K*α radiation
μ = 0.07 mm^−1^
*T* = 296 K
0.30 × 0.12 × 0.10 mm

#### Data collection

Bruker Kappa APEXII CCD diffractometer
Absorption correction: multi-scan (*SADABS*; Bruker, 2005 ▶) *T*_min_ = 0.980, *T*_max_ = 0.993
8641 measured reflections
2066 independent reflections
1075 reflections with *I* > 2σ(*I*)
*R*_int_ = 0.065

#### Refinement

*R*[*F* ^2^ > 2σ(*F* ^2^)] = 0.052
*wR*(*F* ^2^) = 0.142
*S* = 1.01
2066 reflections
139 parameters
H-atom parameters constrained
Δρ_max_ = 0.15 e Å^−3^
Δρ_min_ = −0.13 e Å^−3^

### 

Data collection: *APEX2* (Bruker, 2009 ▶); cell refinement: *SAINT* (Bruker, 2009 ▶); data reduction: *SAINT*; program(s) used to solve structure: *SHELXS97* (Sheldrick, 2008 ▶); program(s) used to refine structure: *SHELXL97* (Sheldrick, 2008 ▶); molecular graphics: *ORTEP-3* (Farrugia, 1997 ▶) and *PLATON* (Spek, 2009 ▶); software used to prepare material for publication: *WinGX* (Farrugia, 1999 ▶) and *PLATON*.

## Supplementary Material

Crystal structure: contains datablocks global, I. DOI: 10.1107/S1600536810031867/hb5606sup1.cif

Structure factors: contains datablocks I. DOI: 10.1107/S1600536810031867/hb5606Isup2.hkl

Additional supplementary materials:  crystallographic information; 3D view; checkCIF report

## Figures and Tables

**Table 1:** Hydrogen-bond geometry (Å, °) *Cg*1 is the centroid of the C10–C13/N2 pyrrol ring.

*D*—H⋯*A*	*D*—H	H⋯*A*	*D*⋯*A*	*D*—H⋯*A*
N2—H2⋯N1^i^	0.86	2.20	3.017 (3)	158
C9—H9⋯*Cg*1^ii^	0.93	2.80	3.606 (3)	145

Symmetry codes: (i) 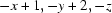; (ii) 
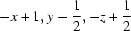
.

**Fig. 1.**
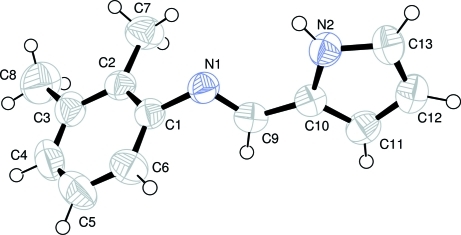
View of (I) with displacement ellipsoids drawn at the 50% probability level.

**Fig. 2.**
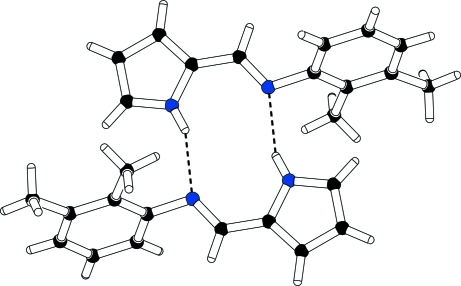
The partial packing of (I), which shows that molecules form dimers.

## supplementary crystallographic information

### Comment

We have reported crystal structures of Schiff bases containing 2,3-dimethylaniline (Tariq *et al.*, 2010), (Sarfraz *et al.*, 2010) and (Hussain *et al.*, 2010*b*) and as a part of this project, we report herein the structure and synthesis of the title compound (I, Fig. 1).

The crystal structure of (II) *i.e.*, *N*^'^-[(*E*)-(1-methyl-1*H*-pyrrol-2-yl)methylidene]benzohydrazide (Hussain *et al.*, 2010*a*) has been published which contain the common 1*H*-pyrrole-2-carbaldehyde moiety as in (I).

In (I), the 2,3-dimethylanilinic group A (C1—C8/N1) and the 1*H*-pyrrole-2-carbaldehyde moiety B (C9—C13/N2) are planar with r. m. s. deviations of 0.0197 and 0.0094 Å, respectively. The dihedral angle between A/B is 70.50 (7)°. The molecules form dimers (Fig. 2) due to intermolecular H-bonding of N—H···N type (Table 1) and complete *R*_2_^2^(10) ring motif (Bernstein *et al.*, 1995). In stabilization of the molecules C—H···π interactions (Table 1) also play important role.

### Experimental

Equimolar quantities of 2,3-dimethylaniline and 1*H*-pyrrole-2-carbaldehyde were refluxed in methanol for 45 min resulting in a clear solution. The solution was kept at room temperature which afforded colourless needles of (I) after 48 h.

### Refinement

The coordinates of H-atoms of water molecule were refined. The H-atoms were positioned geometrically (N–H = 0.86, C–H = 0.93–0.96 Å) and refined as riding with *U*_iso_(H) = *xU*_eq_(C, N), where *x* = 1.5 for methyl and *x* = 1.2 for all other H-atoms.

### Crystal data

**Table:**

C_13_H_14_N_2_	*F*(000) = 424
*M**_r_* = 198.26	*D*_x_ = 1.157 Mg m^−^^3^
Monoclinic, *P*2_1_/*c*	Mo *K*α radiation, λ = 0.71073 Å
Hall symbol: -P 2ybc	Cell parameters from 1075 reflections
*a* = 12.8684 (9) Å	θ = 3.2–25.3°
*b* = 7.1649 (5) Å	µ = 0.07 mm^−^^1^
*c* = 12.9517 (9) Å	*T* = 296 K
β = 107.613 (3)°	Needle, colourless
*V* = 1138.18 (14) Å^3^	0.30 × 0.12 × 0.10 mm
*Z* = 4	

### Data collection

**Table:**

Bruker Kappa APEXII CCD diffractometer	2066 independent reflections
Radiation source: fine-focus sealed tube	1075 reflections with *I* > 2σ(*I*)
graphite	*R*_int_ = 0.065
Detector resolution: 8.10 pixels mm^-1^	θ_max_ = 25.3°, θ_min_ = 3.2°
ω scans	*h* = −15→15
Absorption correction: multi-scan (*SADABS*; Bruker, 2005)	*k* = −8→8
*T*_min_ = 0.980, *T*_max_ = 0.993	*l* = −15→15
8641 measured reflections	

### Refinement

**Table:**

Refinement on *F*^2^	Secondary atom site location: difference Fourier map
Least-squares matrix: full	Hydrogen site location: inferred from neighbouring sites
*R*[*F*^2^ > 2σ(*F*^2^)] = 0.052	H-atom parameters constrained
*wR*(*F*^2^) = 0.142	*w* = 1/[σ^2^(*F*_o_^2^) + (0.0559*P*)^2^ + 0.0942*P*] where *P* = (*F*_o_^2^ + 2*F*_c_^2^)/3
*S* = 1.01	(Δ/σ)_max_ < 0.001
2066 reflections	Δρ_max_ = 0.15 e Å^−^^3^
139 parameters	Δρ_min_ = −0.13 e Å^−^^3^
0 restraints	Extinction correction: *SHELXL97* (Sheldrick, 2008), Fc^*^=kFc[1+0.001xFc^2^λ^3^/sin(2θ)]^-1/4^
Primary atom site location: structure-invariant direct methods	Extinction coefficient: 0.019 (4)

### Special details

**Table:**

Geometry. Bond distances, angles *etc*. have been calculated using the rounded fractional coordinates. All su's are estimated from the variances of the (full) variance-covariance matrix. The cell e.s.d.'s are taken into account in the estimation of distances, angles and torsion angles
Refinement. Refinement of *F*^2^ against ALL reflections. The weighted *R*-factor *wR* and goodness of fit *S* are based on *F*^2^, conventional *R*-factors *R* are based on *F*, with *F* set to zero for negative *F*^2^. The threshold expression of *F*^2^ > σ(*F*^2^) is used only for calculating *R*-factors(gt) *etc*. and is not relevant to the choice of reflections for refinement. *R*-factors based on *F*^2^ are statistically about twice as large as those based on *F*, and *R*- factors based on ALL data will be even larger.

### Fractional atomic coordinates and isotropic or equivalent isotropic displacement parameters (Å^2^)

**Table:**

	*x*	*y*	*z*	*U*_iso_*/*U*_eq_	
N1	0.39549 (16)	0.8356 (3)	0.05111 (16)	0.0532 (8)	
N2	0.62151 (16)	0.9535 (3)	0.15496 (16)	0.0550 (8)	
C1	0.2837 (2)	0.7796 (3)	0.01500 (19)	0.0499 (9)	
C2	0.25093 (19)	0.6419 (4)	−0.06368 (19)	0.0539 (9)	
C3	0.1406 (2)	0.5922 (4)	−0.1006 (2)	0.0659 (11)	
C4	0.0680 (2)	0.6814 (4)	−0.0579 (3)	0.0787 (11)	
C5	0.1015 (2)	0.8157 (4)	0.0209 (3)	0.0834 (14)	
C6	0.2096 (2)	0.8668 (4)	0.0569 (2)	0.0675 (11)	
C7	0.3339 (2)	0.5434 (4)	−0.1043 (2)	0.0766 (11)	
C8	0.0995 (3)	0.4408 (5)	−0.1836 (2)	0.1085 (16)	
C9	0.44692 (19)	0.8048 (3)	0.1505 (2)	0.0516 (9)	
C10	0.55763 (19)	0.8560 (3)	0.20329 (18)	0.0495 (9)	
C11	0.6209 (2)	0.8162 (3)	0.3068 (2)	0.0592 (10)	
C12	0.7242 (2)	0.8895 (4)	0.3205 (2)	0.0642 (10)	
C13	0.7225 (2)	0.9738 (4)	0.2261 (2)	0.0635 (10)	
H2	0.60086	0.99538	0.08967	0.0660*	
H4	−0.00546	0.64985	−0.08297	0.0945*	
H5	0.05137	0.87191	0.04976	0.1003*	
H6	0.23263	0.95956	0.10904	0.0810*	
H7A	0.39874	0.61809	−0.08930	0.1147*	
H7B	0.30476	0.52419	−0.18105	0.1147*	
H7C	0.35134	0.42497	−0.06860	0.1147*	
H8A	0.13149	0.32366	−0.15456	0.1628*	
H8B	0.11924	0.46977	−0.24760	0.1628*	
H8C	0.02160	0.43251	−0.20160	0.1628*	
H9	0.40938	0.74430	0.19188	0.0620*	
H11	0.59856	0.75163	0.35870	0.0711*	
H12	0.78356	0.88212	0.38276	0.0770*	
H13	0.78071	1.03508	0.21258	0.0763*	

### Atomic displacement parameters (Å^2^)

**Table:**

	*U*^11^	*U*^22^	*U*^33^	*U*^12^	*U*^13^	*U*^23^
N1	0.0534 (13)	0.0565 (14)	0.0494 (13)	−0.0073 (10)	0.0151 (10)	−0.0022 (10)
N2	0.0577 (14)	0.0540 (13)	0.0507 (12)	−0.0053 (11)	0.0125 (11)	0.0011 (11)
C1	0.0516 (16)	0.0490 (15)	0.0490 (14)	−0.0008 (12)	0.0153 (12)	0.0051 (13)
C2	0.0564 (17)	0.0560 (17)	0.0510 (15)	−0.0043 (13)	0.0186 (13)	0.0053 (13)
C3	0.0591 (19)	0.072 (2)	0.0619 (18)	−0.0102 (15)	0.0113 (15)	0.0075 (15)
C4	0.0465 (17)	0.093 (2)	0.088 (2)	−0.0013 (17)	0.0074 (16)	0.019 (2)
C5	0.062 (2)	0.087 (2)	0.107 (3)	0.0197 (18)	0.0343 (19)	0.009 (2)
C6	0.0686 (19)	0.0597 (18)	0.0789 (19)	0.0044 (15)	0.0294 (16)	−0.0047 (15)
C7	0.081 (2)	0.078 (2)	0.0773 (19)	−0.0120 (16)	0.0337 (16)	−0.0202 (17)
C8	0.096 (3)	0.131 (3)	0.091 (2)	−0.048 (2)	0.017 (2)	−0.027 (2)
C9	0.0581 (16)	0.0438 (16)	0.0572 (16)	−0.0029 (12)	0.0238 (13)	−0.0030 (13)
C10	0.0542 (15)	0.0424 (15)	0.0523 (16)	−0.0024 (12)	0.0166 (13)	−0.0010 (12)
C11	0.0691 (18)	0.0551 (17)	0.0535 (16)	0.0032 (14)	0.0185 (14)	0.0019 (13)
C12	0.0635 (19)	0.0616 (18)	0.0590 (17)	0.0050 (14)	0.0056 (14)	−0.0032 (15)
C13	0.0525 (17)	0.0635 (18)	0.0692 (18)	−0.0074 (14)	0.0103 (14)	−0.0044 (16)

### Geometric parameters (Å, °)

**Table:**

N1—C1	1.429 (3)	C11—C12	1.390 (4)
N1—C9	1.276 (3)	C12—C13	1.358 (4)
N2—C10	1.366 (3)	C4—H4	0.9300
N2—C13	1.354 (3)	C5—H5	0.9300
N2—H2	0.8600	C6—H6	0.9300
C1—C2	1.389 (3)	C7—H7A	0.9600
C1—C6	1.381 (4)	C7—H7B	0.9600
C2—C7	1.502 (4)	C7—H7C	0.9600
C2—C3	1.400 (4)	C8—H8A	0.9600
C3—C4	1.378 (4)	C8—H8B	0.9600
C3—C8	1.506 (4)	C8—H8C	0.9600
C4—C5	1.374 (5)	C9—H9	0.9300
C5—C6	1.376 (4)	C11—H11	0.9300
C9—C10	1.429 (4)	C12—H12	0.9300
C10—C11	1.372 (3)	C13—H13	0.9300
			
C1—N1—C9	116.4 (2)	C4—C5—H5	120.00
C10—N2—C13	109.3 (2)	C6—C5—H5	120.00
C10—N2—H2	125.00	C1—C6—H6	120.00
C13—N2—H2	125.00	C5—C6—H6	120.00
N1—C1—C2	119.5 (2)	C2—C7—H7A	109.00
N1—C1—C6	119.4 (2)	C2—C7—H7B	109.00
C2—C1—C6	121.1 (2)	C2—C7—H7C	110.00
C1—C2—C3	118.9 (2)	H7A—C7—H7B	109.00
C3—C2—C7	121.1 (2)	H7A—C7—H7C	109.00
C1—C2—C7	120.0 (2)	H7B—C7—H7C	109.00
C2—C3—C8	121.9 (3)	C3—C8—H8A	109.00
C4—C3—C8	119.1 (3)	C3—C8—H8B	109.00
C2—C3—C4	119.0 (3)	C3—C8—H8C	109.00
C3—C4—C5	121.6 (3)	H8A—C8—H8B	109.00
C4—C5—C6	119.7 (3)	H8A—C8—H8C	109.00
C1—C6—C5	119.6 (3)	H8B—C8—H8C	109.00
N1—C9—C10	125.2 (2)	N1—C9—H9	117.00
N2—C10—C11	107.0 (2)	C10—C9—H9	117.00
C9—C10—C11	128.7 (2)	C10—C11—H11	126.00
N2—C10—C9	124.3 (2)	C12—C11—H11	126.00
C10—C11—C12	108.0 (2)	C11—C12—H12	126.00
C11—C12—C13	107.4 (2)	C13—C12—H12	126.00
N2—C13—C12	108.4 (2)	N2—C13—H13	126.00
C3—C4—H4	119.00	C12—C13—H13	126.00
C5—C4—H4	119.00		
			
C9—N1—C1—C2	−116.5 (3)	C1—C2—C3—C8	−178.4 (2)
C9—N1—C1—C6	65.2 (3)	C7—C2—C3—C4	177.5 (3)
C1—N1—C9—C10	−177.7 (2)	C7—C2—C3—C8	−1.0 (4)
C13—N2—C10—C9	−178.2 (2)	C2—C3—C4—C5	−0.9 (5)
C13—N2—C10—C11	0.3 (3)	C8—C3—C4—C5	177.6 (3)
C10—N2—C13—C12	0.0 (3)	C3—C4—C5—C6	1.6 (5)
N1—C1—C2—C3	−178.2 (2)	C4—C5—C6—C1	−1.4 (4)
N1—C1—C2—C7	4.4 (4)	N1—C9—C10—N2	3.4 (4)
C6—C1—C2—C3	0.1 (4)	N1—C9—C10—C11	−174.7 (2)
C6—C1—C2—C7	−177.3 (2)	N2—C10—C11—C12	−0.4 (3)
N1—C1—C6—C5	178.8 (2)	C9—C10—C11—C12	177.9 (2)
C2—C1—C6—C5	0.5 (4)	C10—C11—C12—C13	0.5 (3)
C1—C2—C3—C4	0.1 (4)	C11—C12—C13—N2	−0.3 (3)

### Hydrogen-bond geometry (Å, °)

**Table:**

Cg1 is the centroid of the C10–C13/N2 pyrrol ring.

**Table:**

*D*—H···*A*	*D*—H	H···*A*	*D*···*A*	*D*—H···*A*
N2—H2···N1^i^	0.86	2.20	3.017 (3)	158
C9—H9···Cg1^ii^	0.93	2.80	3.606 (3)	145

Symmetry codes: (i) −*x*+1, −*y*+2, −*z*; (ii) −*x*+1, *y*−1/2, −*z*+1/2.
